# Antihypertensive Effects of an Optimized Aged Garlic Extract in Subjects with Grade I Hypertension and Antihypertensive Drug Therapy: A Randomized, Triple-Blind Controlled Trial

**DOI:** 10.3390/nu15173691

**Published:** 2023-08-23

**Authors:** José C. E. Serrano, Eva Castro-Boqué, Alicia García-Carrasco, María Inés Morán-Valero, Daniel González-Hedström, Marcelino Bermúdez-López, José Manuel Valdivielso, Alberto E. Espinel, Manuel Portero-Otín

**Affiliations:** 1 NUTREN-Nutrigenomics, Department of Experimental Medicine, University of Lleida, 25198 Lleida, Spain; manuel.portero@udl.cat; 2 Vascular and Renal Translational Research Group, Institut de Recerca Biomèdica de Lleida, IRBLleida, 25198 Lleida, Spain; ecastro.lleida.ics@gencat.cat (E.C.-B.); agarcia@irblleida.cat (A.G.-C.); mbermudez@irblleida.cat (M.B.-L.); valdivielso@irblleida.cat (J.M.V.); 3 Pharmactive Biotech S.L.U., 28049 Madrid, Spain; ines.moran@pharmactive.eu (M.I.M.-V.); dgonzalez@pharmactive.eu (D.G.-H.); aespinel@pharmactive.eu (A.E.E.)

**Keywords:** hypertension, aged black garlic, blood pressure, s-allyl-cysteine

## Abstract

The use of garlic (*Allium sativum*) for treating arterial hypertension has been recognized as effective for several decades. However, tolerance to treatment is low, and several technological modifications have been developed to improve its tolerability, such as the aging process at controlled temperature and humidity. This study aims to validate the antihypertensive effects of an optimized extract of aged black garlic with low doses of s-allyl-cysteine (SAC) in a Grade I hypertensive population with drug treatment. A randomized, triple-blind, placebo-controlled parallel trial was developed, where a daily supplementation with 0.25 mg/day of SAC for 12 weeks was performed. A reduction in systolic and diastolic blood pressure of 1.8 mmHg (0.7 to 4.1 95% CI) and 1.5 mmHg (0.3 to 3.0 95% CI), respectively, was observed. Similarly, an increase in blood nitric oxide (10.3 µM, 1.1 to 19.5 95% CI) and antioxidant capacity (7 × 10^−3^ µM TE/min, (1.2 to 13 × 10^−3^ 95% CI) and a reduction in uric acid levels (−0.3 mg/dL, −0.5 to −0.001 95% CI) and ACE activity (−9.3 U/L; −18.4 to −0.4 95% CI) were observed. No changes in endothelial function and inflammatory cytokines were observed. It was concluded that low-dose SAC supplementation in an optimized black-garlic extract allows for an extra-significant reduction in blood pressure in a Grade I hypertensive population receiving drug treatment.

## 1. Introduction

A wealth of evidence exists to demonstrate that lowering blood pressure can substantially reduce premature morbidity and mortality [1]. However, despite the advances in understanding the pathology and the development of drugs to treat hypertension, it is estimated that only a small percentage of the population worldwide (around 32%) can control hypertension [2]. Indeed, resistant hypertension (subjects receiving three or more drugs of different classes at maximally tolerated doses but still have uncontrolled blood pressure) is found in a substantial part of the adult population [3]. The use of food-bioactive compounds and a healthy lifestyle are suitable options for non-pharmacological approaches to improving vascular health [4]. Among them, organosulfur compounds, a class of organic compounds containing sulfur as a critical component of their molecular structure, have been shown to benefit blood pressure [5]. They are found in various foods, such as garlic, onions, and cruciferous vegetables [6].

Several studies have shown that garlic can help lower blood pressure in individuals with hypertension [7]. A mean reduction of around 9 and 6 mmHg in systolic and diastolic blood pressure could be expected due to garlic supplementation in hypertensive subjects (>140/90 mmHg). This effect is thought to be due to the presence of various compounds, such as allicin, alliin, and S-allyl-L-cysteine (SAC), among others, which have potent anti-inflammatory, antioxidant, and vasodilatory properties [8,9,10]. Nevertheless, although garlic is generally well-tolerated in moderate amounts as part of a balanced diet, some individuals may experience adverse effects when consuming large quantities of raw and cooked garlic or taking high doses of garlic supplements. Therefore, technological modifications of raw garlic in different forms, such as smoked garlic, garlic oil macerate, garlic juice, garlic powder, and fermented garlic, have been developed to alleviate these discomforts and increase dietary tolerance.

Aged black garlic (ABG) is a type of modified garlic that contains a unique composition of organosulfur compounds and polyphenols compared to fresh garlic [11]. During aging, the allicin and other sulfur compounds undergo chemical changes that form new compounds, including SAC and melanoidin [12]. These compounds are responsible for ABG’s distinctive taste, aroma, and color. Regarding hypertension, ABG has been found to have potential benefits due to the high levels of SAC, which have been shown to relax blood vessels, improve blood flow, and reduce oxidative stress [13], with a similar magnitude of effects in cardiovascular physiology compared to raw garlic [14].

In this sense, this study aimed to evaluate the antihypertensive effects of a daily intake of a low-dose SAC-optimized ABG extract for 12 weeks in individuals with Grade I hypertension following blood-pressure-reducing drug therapy. For that purpose, a randomized, triple-blind, placebo-controlled parallel trial was developed, where the primary outcome was the reduction in office and home blood pressure. Secondary outcomes such as endothelial function, nitric oxide, inflammatory cytokines, angiotensin-converting enzyme activity, and antioxidant capacity levels in blood were determined to explain the antihypertensive effect of ABG.

## 2. Materials and Methods

### 2.1. Experimental Design

The study was designed as a triple-blind, placebo-controlled, randomized parallel study to determine whether an optimized aged black garlic (ABG) extract could reduce systolic and diastolic blood pressure in subjects with Grade I hypertension. The study consisted of two periods. An initial period called the “Run-in phase”, two weeks in duration, involved all volunteers being treated with a placebo, and blood pressure was monitored daily and served as the basis for the evaluation of future changes in blood pressure. At least five consecutive days of home blood pressure monitoring are required for a reliable diagnosis of home hypertension [15]. Therefore, two weeks of monitoring is adequate to establish volunteers’ basal blood pressure. Similarly, it helped determine volunteers that were non-adherent to the study protocol before the experimental phase. The second period was called the “Experimental phase”. During this phase, the volunteers were randomly divided into groups—Group A and Group L—and were treated for 12 weeks with a placebo or active ingredient, respectively. Treatment time was determined based on previous studies reported in different meta-analyses on garlic and hypertension [16,17].

Volunteers were randomly assigned to each group by sequential number assignment, and they were divided by sex after their acceptance to participate in the study. The study was carried out under the ethical principles established in the latest version of the Declaration of Helsinki and Tokyo for human investigation and following Regulation (EU) 2016/679 of the European Parliament of the Council of 27 April 2016 on Data Protection (RGPD); Law 14/2007 on biomedical research; and Organic Law 3/2018 on the Protection of Personal Data and Guarantee of Digital Rights. Participants provided informed consent before they participated in the study. The study protocol was approved by Hospital Universitari Arnau de Vilanova (CEIC-2435), Lleida, Spain, and the trial is registered at ClinicalTrials.gov NCT04915053. No changes to methods after trial commencement were introduced.

### 2.2. Intervention

The intervention consisted of a daily intake of one tablet after wake-up and before breakfast. Group L received an optimized ABG10+ -extract tablet manufactured by Pharmactive Biotech Products, S.L.U. (Madrid, Spain), according to a proprietary process. The extract was from garlic harvested in the area of Las Pedroñeras (Castilla la Mancha, Spain), and the tablets were manufactured at Instant Procès (La Roca del Vallès, Spain). Each ABG tablet contained 250 mg of ABG extract, which provided 0.25 mg of SAC per tablet plus 300 mg of the excipients shown in Table 1. Group A received a placebo tablet where the ABG extract was replaced by maltodextrin. Both pills were similar in appearance and smell and coded as A or L by the manufacturer. To guarantee blinding, none of the researchers involved in the study knew the composition of each tablet. Participants were instructed to maintain their dietary and physical habits and medical treatments. Changes in antihypertensive drug treatment were asked about at the end of the experimental phase. None of the volunteers reported changes in antihypertensive drug treatment. Volunteer recruitment and trial beginning started in September 2021 and ended in April 2022.

### 2.3. Study Population

Volunteers were recruited in collaboration with the Atherothrombotic Disease Detection and Treatment Unit (UDETMA) of the Hospital Universitari Arnau de Vilanova (Lleida, Spain) from the database of patients with Grade I hypertension. The inclusion criteria included: men and women over 18 years of age with diagnosed hypertension that were under treatment with one or more antihypertensive drugs, excluding ACE inhibitors. Exclusion criteria included suffering from morbid obesity (body mass index > 35 kg/m^2^), serum LDL-cholesterol levels < 115 mg/dL (set point of low or moderate SCORE risk of cardiovascular events), fasting serum glucose levels > 126 mg/dL (cut-off parameter among the criteria of diabetes), anemia (hemoglobin < 13 g/dL in mean and <12 g/dL in women), chronic gastrointestinal disease, being pregnant or intending to become pregnant, lactation, intending to participate or have participated in a clinical trial or nutritional intervention study in the last 30 days before study enrolment, and allergy to garlic.

### 2.4. Sample Size Determination

The sample size was calculated based on the study published by Rothwell et al. [18], in which it was estimated that the within-individual visit-to-visit variability in SBP was between 11.4 and 14.9 mmHg (mean, standard deviation of 13.1 mmHg). The expected outcome after ABG-extract supplementation was a reduction of 9 mmHg in systolic blood pressure, following the results of a recent meta-analysis of garlic-extract intervention in blood pressure management [7]. The number of volunteers needed in each intervention group for a two-sided test, a 0.05 Type I error rate, and an estimated power of 0.90 was 36.

### 2.5. Outcomes

#### 2.5.1. Blood Pressure

Office and home blood pressure were determined by the same oscillometric automatic sphygmomanometers (Medisana BU 512, Medisana GmbH, Neuss, Germany), with a maximum error tolerance for static pressure of ±3 mmHg. Volunteers were seated comfortably in a quiet environment for 5 min before blood pressure measurements. Office and home blood pressure were determined by the average of three recordings of systolic and diastolic blood pressure, obtained at 3 min intervals after subjects had been seated on a chair with their feet on the floor and arms supported at the heart level. For home blood pressure measurements, volunteers were instructed to take measurements in the morning, 15 min after getting up and urinating (if applicable); to sit comfortably; and to take blood pressure three times at 5 min intervals. They were provided with a notebook where they had to record the measurements daily, including the exact time. Additionally, at the time of the appointments, the measurements provided by the volunteer were verified by consulting the memory of the sphygmomanometer and, if necessary, corrected in their notebook based on the time and date of measurement.

#### 2.5.2. Endothelial Function

The EndoPAT™ parameters reactive hyperemia index (RHI) and augmentation index (AI_75_) were assessed to evaluate endothelial function during the experimental period (basal and 12 weeks after the intervention). Briefly, the thermoneutral room temperature was always maintained (24 °C) during the analysis. Any restrictive clothing, watches, or rings that could interfere with blood flow to the arms and fingers were removed from the volunteer. Further, the upper-arm blood pressure cuff was applied snuggly. The volunteer was comfortably lying in the study room and relaxed for at least 15 min before the analysis. The measurement protocol consisted of 1 min of reading, where the correct placement of the sensors and the perceived signal were verified, followed by 5 min of basal conditions, 5 min of occlusion, and 5 min of post-occlusion recording. Occlusion was performed by increasing the blood pressure cuff to a supra-systolic level of at least 60 mmHg above systolic blood pressure and no less than 200 mmHg. A total absence of signal from the occluded hand verified blood-flow cessation. When the appearance of any movement was noted, cuff pressure was increased by an additional 50 mmHg, up to 300 mmHg. RHI and AI_75_ values were automatically calculated by EndoPAT™ 2000 software. Occlusion borders were verified in all measurements, and manual corrections were performed when necessary.

#### 2.5.3. Nitric Oxide

Nitric oxide was determined by nitrate/nitrite concentration using a fluorometric assay kit (Abcam ab65327, Cambridge, UK) following manufacturer instructions. Serum samples were filtered with a 10 kDa Spin column (Abcam ab93349, Cambridge, UK) to deproteinize the samples before the analysis. The analysis consisted of a two-step process: first, nitrate is converted to nitrite by nitrate reductase, and then nitrite reacts with the fluorescent probe 2,3 diaminonaphthalene. A nitrite standard calibration curve was used to compare the fluorescence intensity of samples. The analysis was performed in triplicate in each sample.

#### 2.5.4. Antioxidant Capacity

Antioxidant capacity was measured by the FRAP (ferric reducing antioxidant power) assay. Briefly, 900 μL of the FRAP reagent containing 2,4,6-tri(2-pyridyl)-s-triazine, FeCl_3_, and acetate buffer (300 mM, pH 3.6) was mixed with 90 μL of distilled water and 30 μL of plasma, or sodium phosphate buffer for the blank. Maximum absorbance values of this solution at 595 nm were taken every minute for 30 min at 37 °C, using a Beckman DU640 spectrophotometer (Beckman Instruments Inc., Fullerton, CA, USA). Antioxidant capacity was referenced to standards containing known concentrations of 6-hydroxy-2,5,7,8-tetramethyl chroman-2-carboxylic acid (Trolox) and expressed as μM Trolox Equivalents (TE). Total antioxidant capacity was defined as the slope of the increase in absorbance.

#### 2.5.5. Inflammatory Cytokines

The simultaneous assessment of serum concentrations of IL-2, IL-4, IL-5, IL-8, INF-γ, inducible protein 10 (IP-10), and soluble CD40 ligand (sCD40L) was performed using commercially available multiplex bead-based immunoassay kits (Milliplex MAP Human Cytokine/Chemokine Magnetic Bead Panel, HCYTOMAG-60K, Millipore, Billerica, MA, USA) following manufacturer’s instructions. Briefly, plasma samples (25 μL/well) or standards (25 μL well) were incubated with 25 μL of the pre-mixed bead sets in pre-wetted 96-well microtiter plates at 4 °C overnight. After washing, 25 μL of the fluorescent detection antibody mixture was added for 30 min, and 25 μL of streptavidin-phycoerythrin was added to each well for 30 min at room temperature. A range of 3.2–10,000 pg/mL recombinant cytokines was used to establish standard curves and maximize the assay’s sensitivity and dynamic range. Cytokine levels were determined using a Luminex IS 100 (Luminex, Austin, TX, USA).

#### 2.5.6. Angiotensin-Converting Enzyme (ACE) Activity

ACE activity was determined following the manufacturer’s instruction of Spinreact’s (41205, Sant Esteve de Bas, Spain) quantitative determination kit. Briefly, in this method, the direct substrate *N*-[3-(2-furyl)-acryloyl]-Lphenylalanylglyclglycine (FAPGG) is hydrolyzed to FAP and glycylglycine. The hydrolysis of FAPGG by volunteer serum ACE results in a decrease in absorbance at 340 nm. A standardized calibrator (Spinreact, 1002225, Sant Esteve de Bas, Spain) was employed to estimate ACE activity in samples.

#### 2.5.7. Statistical Analysis

Statistical analysis was performed as per protocol. Primary and secondary parameters were measured at baseline and after 12 weeks of intervention. Data are presented as mean ± standard deviation of the mean. Two-way ANOVA and multiple comparisons, used to analyze the treatment effect, were performed using Sidak’s test. A statistical analysis of initial differences between Groups A and L was performed by a two-tailed unpaired Student’s t-test. A Chi-square test was performed for categorical variables. Correlation analysis between the change in blood pressure and confounding variables was performed by Pearson’s correlation analysis. To determine the evolution in home blood pressure measurements, linear regressions were conducted between the days of treatment (0–84 days) and the mean of the blood pressure taken daily. Differences in slopes were monitored by a two-tailed t-test analysis. In all cases, the cut-off to determine significant differences between groups was set at a *p*-value below 0.05. Statistical analyses and graphs were achieved with GraphPad Prism v6.

## 3. Results

### 3.1. Study Design

**Figure 1:**
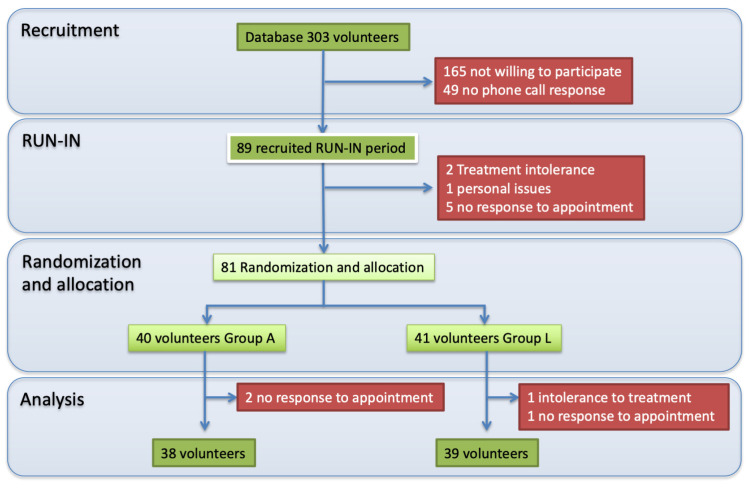
Study and participants flowchart.

The recruitment phase included creating a database of potential volunteers according to the inclusion and exclusion criteria. Of the 303 patients in the database, 89 volunteers showed interest in participating in the study and entered the run-in phase. Eight volunteers did not complete the run-in phase due to placebo intolerances, personal issues, and non-response to the next appointment. Eighty-one volunteers were randomized to participate in one of the two groups of the present study (Group A: Placebo and Group L: Active compound) and started the experimental phase. Finally, 38 and 39 volunteers from Groups A and L completed the experimental phase. The leading causes of withdrawal from the study were non-response to appointments in Groups A and L and intolerance to treatment in Group L, mainly due to gastroesophageal reflux. A flowchart of the study is depicted in Figure 1. Table 2 describes the baseline characteristics of the participants in both groups, where no differences between groups were found.

### 3.2. Compliance and Reported Adverse Events

Treatment compliance was determined from the number of pills taken divided by the total days of the treatment period. Some volunteers took extra treatment days due to volunteer availability adjustments in appointments. The mean treatment days in both groups were 87 ± 5 and 88 ± 5 for groups L and A, respectively. The mean compliance in both groups was above 96%, where no difference in compliance was observed between groups (*p* = 0.4396). No data were excluded from the study.

Four volunteers reported treatment adverse events. Two volunteers from Group L (active compound) and one from Group A (placebo group) reported mild gastric discomfort, while one volunteer from Group A reported muscular cramps.

The treatment blinding of volunteers was executed by asking each participant in the last appointment if they believed there where in the active or placebo group. Moreover, 23% and 45% of volunteers in Groups L and A, respectively, thought they were in the active group. Therefore, it was determined that volunteer treatment blinding was appropriately conducted. Regarding the researcher blinding, none of the researchers were aware of which group the active compound belonged to until the finalization of the statistical analysis.

### 3.3. Blood Pressure, Endothelial Function, and Related Variables

**Figure 2:**
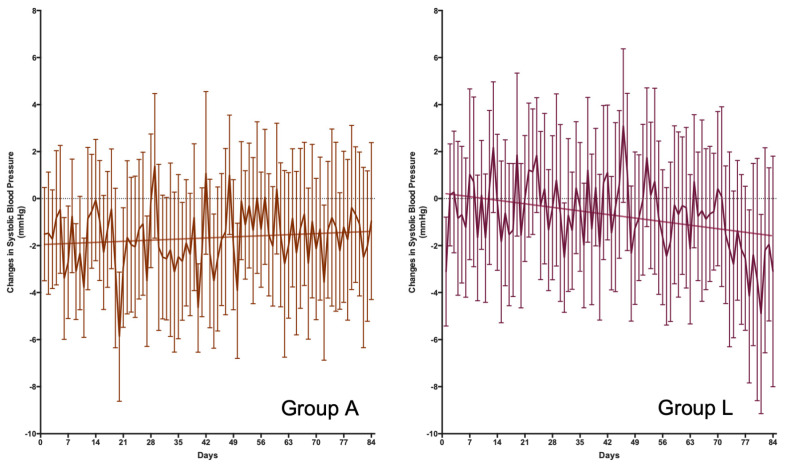
*Daily changes in systolic blood pressure*. Differences in the evolution of blood pressure were observed between Groups A and L (*p* = 0.0033 for the differences in slope). Group A (*n* = 38) did not show a linear regression (*p* = 0.3044), with a mean increase in systolic blood pressure of 6.7 × 10^−3^ mmHg/day after 12 weeks of treatment. Group L (*n* = 39) shows a good fit for a lineal regression (*p* = 0.0021), with a mean decrease in systolic blood pressure of 21.6 × 10^−3^ mmHg/day after 12 weeks of treatment.

**Figure 3:**
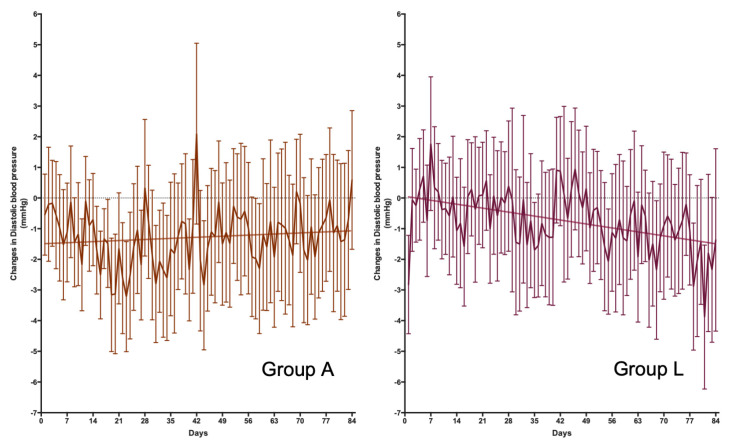
Daily changes in diastolic blood pressure. Differences in the evolution of blood pressure were observed between Groups A and L (*p* = 0.0002 for the differences in slope). Group A (*n* = 38) did not show a linear regression (*p* = 0.2648), with a mean increase in diastolic blood pressure of 5.1 × 10^−3^ mmHg/day after 12 weeks of treatment. Group L (*n* = 39) shows a good fit for a lineal regression (*p* < 0.0001), with a mean decrease in diastolic blood pressure of 18.4 × 10^−3^ mmHg/day after 12 weeks of treatment.

Changes in blood pressure were determined through two methods: the measurements in the office at baseline and 12 weeks and the daily measurement performed by the volunteers at home. The results of the office pressure measurements are shown in Table 3. At the time of the visit, no significant treatment effect was observed on systolic and diastolic blood pressure based on two-way ANOVA analysis. However, post-hoc studies suggest that Group A (placebo) had significantly reduced systolic blood pressure levels. On the other hand, the evolution in the change in daily blood pressure was also analyzed (Figure 2 and Figure 3 for systolic and diastolic blood pressure, respectively). Under these conditions, Group L’s treatment showed a reduction in systolic and diastolic blood pressure levels of about 1.8 and 1.5 mmHg, respectively, after 12 weeks of treatment. The 95% confidence intervals for these changes in blood pressure were from a maximum reduction of 4.1 and 3.0 mmHg and an increase of 0.7 and 0.3 mmHg in systolic and diastolic pressure, respectively.

No changes in endothelial function parameters (RHI and AI_75_ values) were observed in either group. Similarly, no significant differences in the blood lipid profile were observed (Table 3).

Regarding the variables that could explain ABG-extract effects on blood pressure, except for IL-2 levels, there were no significant changes due to treatment in the variables included in Table 3. However, post-hoc analyses suggest that Group L increased nitric oxide, antioxidant capacity measured by the FRAP method, cytokine IP-10 levels, and reduced ACE activity and blood uric acid levels. On the other hand, Group A presented a reduction in the levels of the cytokine sCD40L.

### 3.4. Confounding Variables Analysis

No differences in response to the treatment were observed due to sex (*p*-value of 0.7544 and 0.8484 for systolic and diastolic blood pressure) or the intake of blood pressure-lowering drugs (*p*-value of 0.1183). Nevertheless, it was observed that the volunteers with lower basal values of RHI (r = −0.311 and *p*-value 0.0395) and higher basal levels of the cytokine IP10 (r = 0.0077 and *p*-value 0.0077) in Group L where the ones with a lower response to the treatment.

## 4. Discussion

This study aimed to validate whether daily supplementation with an optimized black-garlic extract could help to control blood pressure in medicated people with Grade I hypertension. The results of this study suggest a drop in systolic and diastolic blood pressure of 1.8 and 1.5 mmHg, respectively, at values measured at home, not observing significant changes in the measurements taken in the office.

Garlic has repeatedly shown a beneficial effect in lowering blood pressure [19]. The hypotensive response has been found in many products obtained from fresh, dried garlic and processed garlic through fermentation or aging. Some meta-analyses and reviews agree with such conclusions [17]. The calculated reduction in blood pressure is higher than the results shown in the current study. However, the conclusion presented in previous meta-analyses and reviews could be biased by notorious design flaws in the selected clinical trials. In some cases, the botanic material remained uncharacterized; in other cases, the active compound was not a defined traditional local product [20], or the study included additional interventions such as dieting.

On the other hand, it has also been shown that a daily ABG-extract supplementation, even at low doses of SAC (0.25 mg/day), induces significant effects on blood pressure control. This last point is critical to highlight since it demonstrates garlic extracts at low doses that are effective without inducing commonly reported side effects [21] or other adverse effects. For example, case reports have highlighted the possibility that garlic use at high doses may cause allergic reactions, the alteration of platelet function and coagulation [22], and gastrointestinal discomfort, among other things. Different sulfur-containing compounds may be responsible for the anticoagulant and antiplatelet effect [23]. Indeed, large quantities of garlic have been discouraged by reputed authorities before surgery or by persons under warfarin or other anticoagulant treatment because it may increase bleeding time. In this study, no changes in the levels of sCD40L have been observed, suggesting no potential effects of ABG consumption on coagulation [24]. Finally, the results of this study showed that only three volunteers reported mild discomfort, which did not interfere with treatment adherence.

The reduction in blood pressure measured at home is considered even more relevant than the measurement in the office since recent meta-analyses have indicated that home blood pressure monitoring better predicts cardiovascular morbidity and mortality than office blood pressure [25]. In this context, it is considered that for every 10 mmHg reduction in blood pressure, the risk of developing cardiovascular events and mortality is reduced by 13% [1]. The drop in blood pressure observed in this study did not reach 10 mmHg. However, it should be considered that even a modest blood pressure reduction can lead to meaningful gains in preventing incidents or recurrent cardiovascular disease [26]. Indeed, as observed in this study, even a 2 mmHg lower usual systolic blood pressure would mean about 10% lower stroke mortality and about 7% lower mortality from ischaemic heart disease or other vascular causes in middle age [27]. Similarly, it should be considered that the volunteers included in this study have a medical history of hypertension of an average of 15 years, where despite the pharmacological treatment, they showed high blood pressure levels. Adding ABG extract to treat arterial hypertension effectively controls blood pressure in a population resistant to drug treatment without inducing side effects.

The changes in blood pressure observed in this study are consistent with previous observations of the effect of the same ABG extract on cardiovascular physiology. A clinical trial in subjects with moderate hypercholesterolemia reported reduced diastolic blood pressure, particularly in men with diastolic blood pressure above 75 mmHg [28], without changes in systolic blood pressure. However, it should be noted that the changes in blood pressure were observed in subjects without hypertension diagnosis and medication. Similarly, in in-vitro studies with rat hearts subjected to ischemia and reperfusion using the Langendorff technique, it was observed that ABG extracts directly increased the release of nitric oxide in aorta segments, inducing a relaxing effect on coronary arteries [11]. The previously described mechanisms of action suggest that the results may be related to the increased relaxation capacity of the endothelial wall. In this sense, it has been observed that ABG could induce its effects through an increase in nitric oxide levels; the antioxidant capacity of the blood to preserve nitric oxide; the reduction of uric acid; and by reducing ACE activity. Other researchers have demonstrated the acute effects of other black-garlic extracts on the relaxation capacity of the arterial wall, consistent with an increase in NO levels [29]. On the other hand, in another study, it was not observed that vasorelaxation effects could be mediated by an increase in H_2_S [30]. Nevertheless, no changes were observed in endothelial-relaxation capacity measured by RHI and AI_75_ indexes in this study. A similar finding is reported with other garlic extracts [31]. Interestingly, the changes in RHI were higher in subjects with higher initial values of RHI, suggesting that the volunteers that presented a reduced vasodilation capacity after hypoxia would not respond to ABG-extract treatment.

Regarding inflammation markers, previous studies suggest that components of ABG extract present anti-inflammatory properties in models of lipopolysaccharide toxin inflammation induction [32]. In this study, the effects on inflammation markers were not significant, possibly due to the low inflammatory status of the volunteers and the low doses of ABG extract used. An increase in levels of IL-2 was observed at the end of the experimental period; however, this modification was observed in both groups, and it was considered a time-dependent effect. Similarly, there was an increase in the levels of IP-10 in the ABG-extract treatment group, although the observed levels were in the range observed in healthy people (2205–4700 pg/mL) [33].

It is suggested that combination therapy is more effective than increasing doses of a single blood-pressure-reducing drug [34]. ABG-extract supplementation could be involved in several controlling mechanisms. The reduction of oxidative stress and the antihypertensive effect via ACE inhibition have been proposed as well as H_2_S-regulating and NO-stimulating properties [35]. The results from this study did not suggest any difference in blood pressure improvement in the volunteers with combination therapy (two or more drugs) compared to monotherapy. However, due to the wide variability of medications used by the volunteers, it is difficult to determine if there is an interaction between the ABG extract and the type and number of medications used. Similarly, no ABG extract–dietary interaction was observed. No correlation was observed between the intake of dietary raw garlic and onion and the change in blood pressure (with the change in systolic blood pressure *p* = 0.6656 and *p* = 0.2638 for garlic and onion, respectively; and for the difference in diastolic blood pressure *p* = 0.6392 and *p* = 0.5370 for garlic and onion, respectively).

This study has some limitations. Although a significant reduction in blood pressure was observed, the time of the day of administration can be optimized. Treatment-time differences affect the beneficial and adverse effects of different blood pressure-reducing drugs [36]. For example, α-adrenoceptor antagonists and β-blockers seem more effective upon awaking. In contrast, calcium-channel blockers, angiotensin-converting enzyme inhibitors, angiotensin II receptor blockers, and diuretics have been reported to be more effective at bedtime. How ABG extract improves blood pressure cannot be attributed to a unique mechanism of action, as described with drug therapy. Therefore, it is difficult to ascertain the ideal time and number of administration doses. For that purpose, ambulatory blood pressure monitoring should be employed in further studies to validate the ABG effect in a 24 h period and at different amounts and administration times. Similarly, this study lacks a dose-effect analysis. Previous studies have reported a dose-dependent effect, ranging between 240 and 960 mg of SAC/day [37]. Nevertheless, in this last study, it was observed that higher doses of an aged garlic extract induced a lower reduction in blood pressure. This could be linked to poorer compliance with and lesser tolerability of the extract to a different composition of aged garlic and ABG in bioactive compounds. In this sense, an analysis of the effectiveness of lower doses of ABG extract, its treatment optimization in the daytime, and the number of doses to be employed is desirable.

## 5. Conclusions

Based on the results obtained in this study, it can be concluded that daily supplementation with ABG extracts with low doses of SAC (0.25 mg/day) can induce significant reductions in blood pressure in individuals with Grade I hypertension and antihypertensive drug therapy.

## Figures and Tables

**Table 1:** Detailed composition of tablets employed in the study.

	Group A Placebo (mg per Tablet)	Group L ABG Extract (mg per Tablet)
ABG extract	-	250
Maltodextrin	250	-
Microcrystalline cellulose	90	90
Dicalcic phosphate	157	157
Sodium croscarmellose	10	10
Magnesium stearate	7	7
Sodium Alginate	3.06	3.06
Stearic acid	0.03	0.03
Oleic acid	1.54	1.54
Medium-chain triglycerides	2.80	2.80
Ethylcellulose	13.17	13.17
Hydroxypropyl methylcellulose	4.86	4.86
Hydroxypropyl cellulose	4.86	4.86
Talc	2.88	2.88
Titanium oxide	1.80	1.80
Garlic flavor	1.00	1.00

**Table 2:** Baseline characteristics of participants by group allocation. Data are reported as mean and standard deviation. Statistical differences between groups were analyzed by two-tailed unpaired Student *t*-tests. *p*-values below 0.05 were considered as a statistical difference.

	Group A (Placebo)	Group L (ABG Extract)	*p*-Value
No. of volunteers	38	39	
Gender	Male: 20; Female: 18	Male: 20; Female 19	0.9057
Age (years)	64.1 ± 5.9	63.7 ± 5.8	0.8018
Systolic blood pressure (mmHg)	145.5 ± 17.2	148.8 ± 22.9	0.4876
Diastolic blood pressure (mmHg)	85.3 ± 10.8	85.7 ± 11.2	0.8765
Pulse pressure (mmHg)	60.0 ± 12.9	62.7 ± 15.7	0.4300
Years of diagnosis	15.4 ± 8.5	14.8 ± 11.2	0.8192
Antihypertensive drug number			
1 type	13	13	0.6219
Two types	13	10	
Three or more types	12	16	
Antihypertensive drug type			
Thiazides	25	27	0.9811
β-blockers	11	14	
Angiotensin II receptor blocker	15	16	
Calcium channel blockers	14	17	
Other types of drugs			
Statins	15	17	0.7826
Benzodiazepines	10	9	
Anticoagulants	8	10	
Proton pump inhibitors	12	8	
Nonsteroidal anti-inflammatory drug	14	10	
Antidiabetic	2	4	
Treatment adherence	96.7 ± 4.7	97.8 ± 6.8	0.4396
Minimum	85%	72%	
Maximum	108%	112%	
Treatment blinding			
Yes/No	17/21	9/30	0.0559

**Table 3:** Changes in blood pressure, endothelial function, and related variables. Data are reported as mean and standard deviation. Statistical differences between groups were analyzed by repeated measures of two-way ANOVA (Basal-12 weeks) and Sidak’s multiple comparison tests to determine differences between time in both groups. *p*-values below 0.05 were considered as a statistical difference.

	Group A (Placebo) (*n* = 38)			Group L (ABG Extract) (*n* = 39)			
	Basal	12 Weeks	Sidak’s *p*-Value	Basal	12 Weeks	Sidak’s *p*-Value	Two-Way ANOVA *p*-Value
Blood pressure (office)							
Systolic (mmHg)	144 ± 13	138 ± 14	0.0169	146 ± 21	143 ± 18	0.3011	0.3011
Diastolic (mmHg)	85 ± 7	83 ± 10	0.2497	85 ± 10	83 ± 9	0.2889	0.9219
Pulse pressure (mmHg)	59 ± 12	55 ± 14	0.0632	61 ± 16	60 ± 14	0.6126	0.2011
Endothelial function							
RHI	2.059 ± 0.414	2.033 ± 0.597	0.9583	2.016 ± 0.569	2.068 ± 0.728	0.8276	0.9753
AI_75_	30.4 ± 14.9	26.7 ± 17.2	0.1767	25.0 ± 17.0	21.1 ± 15.1	0.1139	0.1185
Blood lipid profile							
Total cholesterol (mg/dL)	188 ± 43	188 ± 43	>0.9999	209 ± 40	209 ± 39	0.9984	0.9759
Triacylglycerides (mg/dL)	107 ± 53	107 ± 55	0.9986	122 ± 53	117 ± 43	0.8725	0.1394
HDL-cholesterol (mg/dL)	58 ± 15	58 ± 15	0.9939	59 ± 17	61 ± 21	0.8892	0.4172
Inflammatory cytokines							
IL-2 (pg/mL)	0.190 ± 0.657	0.223 ± 0.790	0.9021	0.894 ± 1.844	1.022 ± 2.043	0.2236	0.0282
IL-4 (pg/mL)	113 ± 541	121 ± 566	0.4704	213 ± 708	215 ± 705	0.9556	0.5079
IL-5 (pg/mL)	3.8 ± 10.5	3.6 ± 10.4	0.8234	6.4 ± 16.1	6.5 ± 16.0	0.9021	0.3859
IL-8 (pg/mL)	20.0 ± 29.8	21.6 ± 33.7	0.2054	25.0 ± 31.6	26.7 ± 33.0	0.2018	0.4948
IP-10 (pg/mL)	490 ± 252	446 ± 231	0.3753	450 ± 202	539 ± 332	0.0218	0.6238
sCD40L (pg/mL)	3856 ± 3927	2322 ± 2512	0.0543	3742 ± 3001	3211 ± 3337	0.6710	0.4978
Other variables							
Nitric oxide (µM)	88 ± 23	90 ± 27	0.9188	87 ± 19	97 ± 31	0.0254	0.5101
ACE activity (U/L)	78 ± 44	71 ± 41	0.1495	78 ± 40	69 ± 38	0.0398	0.9560
Uric acid (mg/dL)	5.6 ± 1.4	5.6 ± 1.3	0.9987	6.0 ± 1.4	5.7 ± 1.3	0.0483	0.3986
FRAP (µM TE/min)	0.111 ± 0.034	0.113 ± 0.029	0.6479	0.105 ± 0.036	0.113 ± 0.043	0.0161	0.6972

## Data Availability

The data presented in this study are available on request from the corresponding author.

## References

[1] Ettehad D., Emdin C.A., Kiran A., Anderson S.G., Callender T., Emberson J., Chalmers J., Rodgers A., Rahimi K. (2016). Blood Pressure Lowering for Prevention of Cardiovascular Disease and Death: A Systematic Review and Meta-Analysis. Lancet.

[2] Chow C.K., Teo K.K., Rangarajan S., Islam S., Gupta R., Avezum A., Bahonar A., Chifamba J., Dagenais G., Diaz R. (2013). Prevalence, Awareness, Treatment, and Control of Hypertension in Rural and Urban Communities in High-, Middle-, and Low-Income Countries. JAMA.

[3] Lamirault G., Artifoni M., Daniel M., Barber-Chamoux N., Nantes University Hospital Working Group on Hypertension (2020). Resistant Hypertension: Novel Insights. Curr. Hypertens. Rev..

[4] Borghi C., Cicero A.F.G. (2017). Nutraceuticals with a Clinically Detectable Blood Pressure-Lowering Effect: A Review of Available Randomized Clinical Trials and Their Meta-Analyses. Br. J. Clin. Pharmacol..

[5] Vazquez-Prieto M.A., Miatello R.M. (2010). Organosulfur Compounds and Cardiovascular Disease. Mol. Aspects Med..

[6] Kris-Etherton P.M., Hecker K.D., Bonanome A., Coval S.M., Binkoski A.E., Hilpert K.F., Griel A.E., Etherton T.D. (2002). Bioactive Compounds in Foods: Their Role in Preventing Cardiovascular Disease and Cancer. Am. J. Med..

[7] Ried K. (2016). Garlic Lowers Blood Pressure in Hypertensive Individuals, Regulates Serum Cholesterol, and Stimulates Immunity: An Updated Meta-Analysis and Review. J. Nutr..

[8] Piragine E., Citi V., Lawson K., Calderone V., Martelli A. (2022). Regulation of Blood Pressure by Natural Sulfur Compounds: Focus on Their Mechanisms of Action. Biochem. Pharmacol..

[9] Recinella L., Chiavaroli A., Masciulli F., Fraschetti C., Filippi A., Cesa S., Cairone F., Gorica E., De Leo M., Braca A. (2021). Protective Effects Induced by a Hydroalcoholic *Allium sativum* Extract in Isolated Mouse Heart. Nutrients.

[10] Recinella L., Gorica E., Chiavaroli A., Fraschetti C., Filippi A., Cesa S., Cairone F., Martelli A., Calderone V., Veschi S. (2022). Anti-Inflammatory and Antioxidant Effects Induced by *Allium sativum* L. Extracts on an Ex Vivo Experimental Model of Ulcerative Colitis. Foods.

[11] García-Villalón A.L., Amor S., Monge L., Fernández N., Prodanov M., Muñoz M., Inarejos-García A.M., Granado M. (2016). In Vitro Studies of an Aged Black Garlic Extract Enriched in S-Allylcysteine and Polyphenols with Cardioprotective Effects. J. Funct. Foods.

[12] Lu X., Li N., Qiao X., Qiu Z., Liu P. (2017). Composition Analysis and Antioxidant Properties of Black Garlic Extract. J. Food Drug Anal..

[13] Liu J., Zhang G., Cong X., Wen C. (2018). Black Garlic Improves Heart Function in Patients With Coronary Heart Disease by Improving Circulating Antioxidant Levels. Front. Physiol..

[14] Czompa A., Szoke K., Prokisch J., Gyongyosi A., Bak I., Balla G., Tosaki A., Lekli I. (2018). Aged (Black) versus Raw Garlic against Ischemia/Reperfusion-Induced Cardiac Complications. Int. J. Mol. Sci..

[15] Groenland E.H., Bots M.L., Visseren F.L.J., McManus R.J., Spiering W. (2022). Number of Measurement Days Needed for Obtaining a Reliable Estimate of Home Blood Pressure and Hypertension Status. Blood Press..

[16] Ried K. (2020). Garlic Lowers Blood Pressure in Hypertensive Subjects, Improves Arterial Stiffness and Gut Microbiota: A Review and Meta-Analysis. Exp. Ther. Med..

[17] Wang H.P., Yang J., Qin L.Q., Yang X.J. (2015). Effect of Garlic on Blood Pressure: A Meta-Analysis. J. Clin. Hypertens..

[18] Rothwell P.M., Howard S.C., Dolan E., O’Brien E., Dobson J.E., Dahlöf B., Sever P.S., Poulter N.R. (2010). Prognostic Significance of Visit-to-Visit Variability, Maximum Systolic Blood Pressure, and Episodic Hypertension. Lancet.

[19] Xiong X.J., Wang P.Q., Li S.J., Li X.K., Zhang Y.Q., Wang J. (2015). Garlic for Hypertension: A Systematic Review and Meta-Analysis of Randomized Controlled Trials. Phytomedicine.

[20] Nakasone Y., Nakamura Y., Yamamoto T., Yamaguchi H. (2013). Effect of a Traditional Japanese Garlic Preparation on Blood Pressure in Prehypertensive and Mildly Hypertensive Adults. Exp. Ther. Med..

[21] Borrelli F., Capasso R., Izzo A.A. (2007). Garlic (*Allium sativum* L.): Adverse Effects and Drug Interactions in Humans. Mol. Nutr. Food Res..

[22] Vaes L.P.J., Chyka P.A. (2000). Interactions of Warfarin with Garlic, Ginger, Ginkgo, or Ginseng: Nature of the Evidence. Ann. Pharmacother..

[23] Ryu J.H., Kang D. (2017). Physicochemical Properties, Biological Activity, Health Benefits, and General Limitations of Aged Black Garlic: A Review. Molecules.

[24] Pamukcu B., Lip G.Y.H., Snezhitskiy V., Shantsila E. (2011). The CD40-CD40L System in Cardiovascular Disease. Ann. Med..

[25] Bliziotis I.A., Destounis A., Stergiou G.S. (2012). Home versus Ambulatory and Office Blood Pressure in Predicting Target Organ Damage in Hypertension: A Systematic Review and Meta-Analysis. J. Hypertens..

[26] Canoy D., Nazarzadeh M., Copland E., Bidel Z., Rao S., Li Y., Rahimi K. (2022). How Much Lowering of Blood Pressure Is Required to Prevent Cardiovascular Disease in Patients With and Without Previous Cardiovascular Disease?. Curr. Cardiol. Rep..

[27] Lewington S., Clarke R., Qizilbash N., Peto R., Collins R. (2002). Age-Specific Relevance of Usual Blood Pressure to Vascular Mortality: A Meta-Analysis of Individual Data for One Million Adults in 61 Prospective Studies. Lancet.

[28] Valls R.M., Companys J., Calderón-Pérez L., Salamanca P., Pla-Pagà L., Sandoval-Ramírez B.A., Bueno A., Puzo J., Crescenti A., Del Bas J.M. (2022). Effects of an Optimized Aged Garlic Extract on Cardiovascular Disease Risk Factors in Moderate Hypercholesterolemic Subjects: A Randomized, Crossover, Double-Blind, Sustainedand Controlled Study. Nutrients.

[29] Baik J.S., Min J.H., Ju S.M., Ahn J.H., Ko S.H., Chon H.S., Kim M.S., Shin Y. (2022). Il Effects of Fermented Garlic Extract Containing Nitric Oxide Metabolites on Blood Flow in Healthy Participants: A Randomized Controlled Trial. Nutrients.

[30] Leitão R., de Oliveira G.V., Rezende C., Volino-Souza M., Mesquita J., de Carvalho L.L., Alvares T.S. (2022). Improved Microvascular Reactivity after Aged Garlic Extract Intake Is Not Mediated by Hydrogen Sulfide in Older Adults at Risk for Cardiovascular Disease: A Randomized Clinical Trial. Eur. J. Nutr..

[31] Gruenwald J., Bongartz U., Bothe G., Uebelhack R. (2020). Effects of Aged Garlic Extract on Arterial Elasticity in a Placebo-Controlled Clinical Trial Using EndoPAT^TM^ Technology. Exp. Ther. Med..

[32] Kim D.G., Kang M.J., Hong S.S., Choi Y.H., Shin J.H. (2017). Antiinflammatory Effects of Functionally Active Compounds Isolated from Aged Black Garlic. Phytother. Res..

[33] Kim H.O., Kim H.S., Youn J.C., Shin E.C., Park S. (2011). Serum Cytokine Profiles in Healthy Young and Elderly Population Assessed Using Multiplexed Bead-Based Immunoassays. J. Transl. Med..

[34] Paz M.A., De-La-Sierra A., Sáez M., Barceló M.A., Rodríguez J.J., Castro S., Lagarón C., Garrido J.M., Vera P., Coll-De-Tuero G. (2016). Treatment Efficacy of Anti-Hypertensive Drugs in Monotherapy or Combination: ATOM Systematic Review and Meta-Analysis of Randomized Clinical Trials According to PRISMA Statement. Medicine.

[35] Shouk R., Abdou A., Shetty K., Sarkar D., Eid A.H. (2014). Mechanisms Underlying the Antihypertensive Effects of Garlic Bioactives. Nutr. Res..

[36] Smolensky M.H., Hermida R.C., Ayala D.E., Tiseo R., Portaluppi F. (2010). Administration-Time-Dependent Effects of Blood Pressure-Lowering Medications: Basis for the Chronotherapy of Hypertension. Blood Press. Monit..

[37] Ried K., Frank O.R., Stocks N.P. (2013). Aged Garlic Extract Reduces Blood Pressure in Hypertensives: A Dose-Response Trial. Eur. J. Clin. Nutr..

